# Ficolin-A Enhances Inhibition of the C-Terminal 19 kDa Region of Merozoite Surface Protein-1 of *Plasmodium berghei* Using Test In Vivo

**Published:** 2013

**Authors:** F Chen, Q Liu, Y Xue, YH Huang, FY Huang, Y Lin, GH Tan, J Zhou

**Affiliations:** 1 The Faculty of Life Sciences, Hubei University, 368 Youyi Road, Wuchang, Wuhan 430062, China; 2 Hainan Provincial Key Laboratory of Tropical Medicine, Pharmacy School, Hainan Medical College, Haikou 571199, China; 3 Lab of Medical Engineering, College of Medical Technology and Engineering, Henan University of Science and Techology, Luoyang 471003, China; 4 Wuhan Tuberculosis Dispensary, 28 Baofeng Road, Qiaokou, Wuhan, 430030, China; † Fan Chen and Qiang Liu are co-primary authors

**Keywords:** Ficolin-A, *Plasmodium berghei*, MSP1_19_

## Abstract

**Background:**

Malaria remains a serious public health problem with significant morbidity and mortality. This study was conducted to identify whether ficolin-A could play an active role of against malaria infection.

**Methods:**

The function of ficolin-A was analyzed in mouse model. The open reading frame of ficolin-A was cloned from the liver of new born C57BL/6 mice by RT-PCR and then inserted into the expression vector of eukaryon to construct pVAX1-ficolin-A plasmid. Meanwhile, the open reading frame of the 19-kDa fragment of merozoite surface protein-1 of *Plasmodium berghei* (MSP1_19_) was cloned and then the expression vector of eukaryon, pVAX1- MSP1_19_ was constructed. Both recombinant vectors were used in the mouse model of infection by *Plasmodium berghei*.

**Results:**

pVAX1-ficolin-A alone could not significantly suppress parasite density and prolong survival time of infection mice; however, when injected pVAX1-ficolin-A and pVAX1-MSP1_19_ together, the percent of invasion by Plasmodium was decreased (from 43.78% to 22.23% at 10 day after infection, compared to vector) and the survival time was prolonged significantly in the infection mouse model (*P*=0.01).

**Conclusion:**

Ficolin-A can enhance the immunoprotection of MSP1_19_, it implies ficolin-A may be used as immunoenhancer in the study of vaccine defending malaria.

## Introduction

Malaria remains one of the world's major health problems, causing nearly a million deaths per year (1). Vaccination has been considered as an approach that will complement other strategies for the prevention of this disease. Protection against infection lies on the host's ability to identify and eliminate pathogens while preserving its own integrity. To fulfill this challenge, hosts have evolved two complementary systems, innate immunity and adaptive immunity. The innate system not only represents the first line of defense against pathogens, but also stimulates and orientates the adaptive response that then provides a delayed but memorized response to infection (2). Ficolins are a group of proteins mainly consisting of collagen-like and fibrinogen-like domains and thought to play a role in innate immunity via their carbohydrate- binding activities (3, 4). Three types of human ficolins have been identified: L-ficolin, H-ficolin and M-ficolin, which act as opsonins and lead to complement activation (5, 6). L-ficolin has been demonstrated could inhibit influenza A virus infection both in vitro and in vivo (7).Two types of ficolins have been identified in mice, ficolin A, and ficolin B (8, 9). Ficolin A is expressed mainly in the liver and presents in the circulation, ficolin B was mainly expressed in a myeloid cell lineage in bone marrow but has not been isolated at the protein level yet (10). Ficolin-A plays a crucial role in host defense as a pathogen-associated molecular patterns (PAMPs) recognition molecule, which is executed through the lectin complement pathway. It has demonstrated significant inhibition of *Staphylococcus aureus* growth in mouse model (11). However, little is known about the function of ficolin-A in defense Plasmodium infection.

Many malaria vaccine candidates have been developed, and some of them are being tested in ongoing clinical trials (12, 13). Among these antigens, Merozoite surface protein 1 (MSP1) is a leading malaria vaccine candidate antigen (14). MSP1 contains many fragments, it is produced during schizogony and merozoite maturation, and only the C terminal 19-kDa fragment of MSP-1 (MSP1_19_) remains on the merozoite surface during erythrocyte invasion and therefore is an ideal target for blocking parasite invasion into the erythrocyte (15). MSP1_19_ is highly conserved and composed of two epidermal growth factor-like domains which contain protective epitopes (16, 17). There are considerable evidences that the MSP-1 19-kDa antigen of protozoan parasites is the target of protective immune response. Immunization with recombinant MSP1_19_ of protozoan parasites formulated with Freund's adjuvant or as a GST(glutathione S-transferas) fusion protein produced can protect monkeys or mice, respectively, against challenge infection (18, 19). However, such protection requires formulations unacceptable for human use because of their high toxicity and adverse effects. Single MSP1_19_ could not form protection antibody titer; the mechanism of action of these antibodies is unknown. Therefore, MSP1_19_ is an ideal protein to clarity whether ficonlin-A play an active role in Plasmodium infection model.

This study was conducted to identify whether ficolin-A could play an active role of against malaria infection.

## Materials and Methods

### Mice and parasites

Specific pathogen-free (SPF) newborn C57BL/6J mice, female BALB/c mice between 5 and 6 weeks of age were purchased from the Experimental Animal Center of Hainan Province, People's Republic of China. The mice were housed in macrolon cages in a laminar flow cabinet and provided with ovalbumin-free food and water *adlibitum*. Animals were handled and treated in accordance with the guidelines of Dutch Committee on Animal Experimentations. *Plasmodium berghei*, NK65, a lethal murine malaria parasite, was maintained in our laboratory and used for challenge infection. Experiments were conducted under a protocol approved by the Institutional Animal Care and Use Committee of Hainan Provincial Key Laboratory of Tropical Medicine.

### Preparation of recombinant ficolin-A

Total RNA was extracted from newborn C57BL/6J mice liver, and reverse-transcribed using Superscript III (Invitrogen, Carlsbad, CA). PCR was performed using the cDNA as template to amplify ficolin-A cDNA fragment covering nucleotides 91–1040 (GeneBank031348), by using a primer pair (5′- CGGATCCATATGCAGTGGCCTACGC-3′and5′-CGAATTCGAGACTGGGGCACCTTA -3′, where the underlines denote the engineered restriction sites, B*am*HI and E*co*RI, respectively). The resulting PCR products were cloned into pVAX1 (Invitrogen, Carlsbad, CA) and sequenced.

### Generation recombinant MSP1_19_

MSP1_19_ gene sequences were obtained by PCR amplification using Platinum *Taq* DNA polymerase (TaKaRa). Template for the amplifications were obtained from *Plasmodium berghei* blood stages, the MSP1_19_ fragment, containing nucleotides 5023–5319 (GeneBank, AF187232), amplified by using a primer pair (CGGGATCCATGCTTAATATGGAT and CGGAATTCTTATGCATTAGGGGT), where the underlines denote the engineered restriction sites, B*am*HI and E*co*RI, respectively. Fragments were cloned into pVAX1 (Invitrogen) and sequenced.

### Mammalian cell transient transfection with pVAX1-ficolin-A and pVAX1- MSP1_19_

Plasmids were tested for expression in COS7 cells prior to use in animals. For transient transfection experiments, freshly grown COS7 cells were seeded at 2×10^5^ cells per 35mm tissue culture dish. Cells were grown in RPMI 1640 (Invitrogen, Carlsbad, CA, USA) containing 10% FCS, 2mM glutamine, 100 U/ml penicillin, and 100ug/ml streptomycin. COS7 cells were then incubated in 5% CO2 until 80% confluent. Plasmids expressing ficolin-A and MSP1_19_ were purified using endotoxinfree DNA extraction kit (Qiagen). Four µg of plasmid DNA was used to transfect COS7 cells respectively, using Lipofectin 2000 (Invitrogen) according to the manufacturer's instructions. Media was changed 24h after transfection. Cells were harvested 48h post-transfection and suspended in PBS. The supernatant was then collected and subjected to Western blotting.

### Western blots

Whole cell lysates were prepared in lysis buffer (150 mM NaCl, 50 mM Tris–HCl (pH 7.4), COS7 supernatants were fractionated by SDS-PAGE on 12% (v/v) polyacrylamide gels under reducing conditions and transferred electrophoretically to nitrocellulose membranes. The membranes were then blocked in 5% milk powder overnight at 4 °C. The membranes were probed with rabbit anti-ficolin A or anti- Plasmodium rabbit polyclonal antibodies in PBS containing 0.1% Tween-20 respectively. After washing, the filters were further incubated with horseradish peroxidase (HRP)-conjugated anti-rabbit IgG as a second antibody and blots were developed by diaminobenzidine tetrahydrochloride (DAB) detection (Pierce).

### Immunization protocol

The plasmid (pVAX1-ficolin-A and pVAX1- MSP1_19_) was propagated in *Escherichia coli* (DH5α). Large-scale purification of the expression vector was conducted with Endo Free Plasmid Giga kits (Qiagen, Hilden, Germany) according to the manufacturer's protocol. The plasmid DNA was stored in endotoxin-free H_2_O at -20°C. All mice were female BALB/c mice, and 5–6 weeks of age at the time of first vaccination. Five groups were set: PBS, pVAX1, pVAX1-Ficolin-A, pVAX1-ficolin-A and pVAX1- MSP1_19,_ pVAX1- MSP1_19,_ each group include eight mice. Intramuscular (IM) DNA plasmids were delivered into the *tibialis anterior* muscle (100µg total) in 100µl PBS by the needle injection. All prime mice (including vector and PBS controls) initially received three immunizations at 2-week intervals (via IM routes).

### Challenge infection

Blood from an infected mouse with *P. berghei* NK65 was taken and immediately diluted in PBS to give the lethal dosage (2×10^6^ infected RBC per dose). Mice were infected by intraperitoneal injection at day 0 (Two weeks after last immunization).

### Parasitemia measurements and analyze Survival time

To evaluate the effect of immunization, infection levels were assessed by Giemsa staining of tail smears, and examined by microscopy. The number of newly invaded ring stages was counted, and invasion was expressed as percent invasion calculated using the formula *I*/(*I+U*)×100, where *I* is the number of erythrocytes infected with ring stages in one visual field, *U* is the number of uninfected erythrocytes in the same visual field. Fifty fields of views were counted in each smear, then mean value and standard error analyzed by SPSS 13.0. Three mice were selected randomly from each group, and three smears were prepared for each mouse. Parasitemia assessed on day 2, 4, 6, 8, 10, respectively, through the period of crisis of parasitemia. Mice were feed till 30 days, the survival mice were sacrificed and survival time was analyzed used by Kaplan-Meier (SPSS 13.0).

### Statistical analysis

Data were expressed as mean ± standard deviation SD) and analyzed by one-way ANOVA and q test using SPSS 13.0. A *P* value less than 0.05 was considered statistically significant.

## Results

### Clone and express of ficolin-A and MSP1_19_

**Fig. 1:**
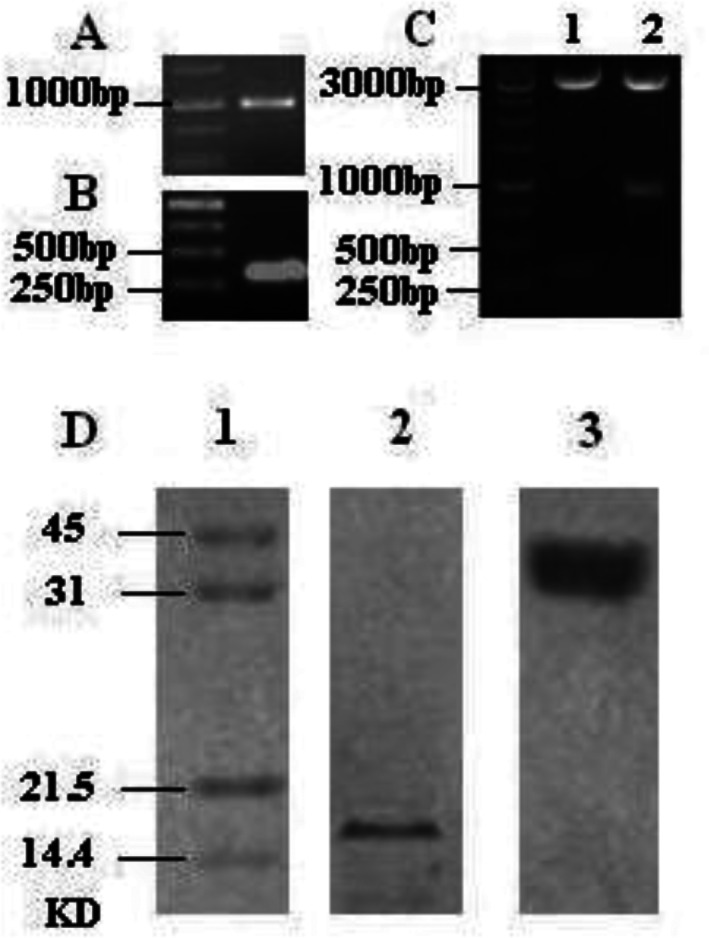
Generation and characterization of recombinant ficolin-A and MSP1_19_. (A) Got the gene of ficolin-A by RT-PCR; (B) Amplification the gene of MSP1_19_ by PCR; (C) Identified the recombinant plasmids, pVAX1- MSP1_19_ and pVAX1-Ficolin-A, lane 1 and 2 respectively, digested by B*amHI* and E*coRI*. (D) Western blotting analysis the expression of pVAX1- MSP1_19_ and pVAX1-ficolin-A in COS7 cells, lane 2 and 3 represent MSP1_19_ and ficolin-A proteins respectively

The open reading frame of ficolin-A was obtained by RT-PCR and MSP1_19_ was amplified by PCR respectively (Fig. 1a, 1b). Both have the same restriction sites, *BamHI* and *EcoRI*. The product of PCR was purified and digested by restriction enzymes, so as the vector of pVAX1. After gel electrophoresis, purification and ligation, plasmids were transformed into *E. coli* DH5α, and positive clone were identified (Fig. 1c). The recombinant plasmids, named pVAX1-ficolin-A and pVAX1- MSP1_19_, were used in the following study. To detect whether the two plasmids can express in eukaryon, pVAX1-ficolin-A and pVAX1- MSP1_19_ were transfected COS7 cells, the results suggest both gene express exactly in eukaryon (Fig. 1d).

### Test of inhibition invasion

**Fig. 2:**
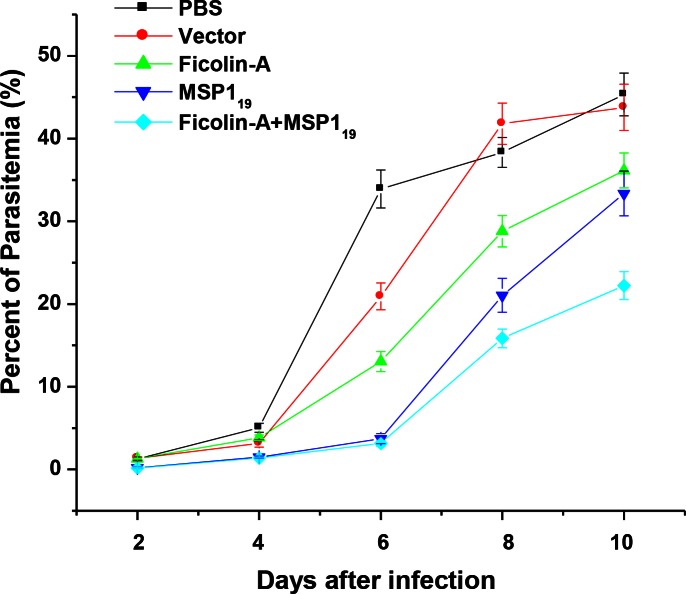
The percent of invasive *Plasmodium* was counted on five groups. Mean value and standard error was marked on 2, 4, 6, 8, 10 days after infection, image automatic generation by Origin 6.0

To evaluate the ability of ficolin-A anti-infection by *Plasmodium*, the percent of invasion erythrocyte by Plasmodium was detected. The percent of invasion for five groups were counted at 2, 4, 6, 8, 10 day, respectively. At 2, 4 day after infection, the percent of invasion for five groups showed approximate data. At 6, 8, 10 day after infection, the percent of invasion were different rapidly, the group of PBS was 33.9%, 38.33%, 45.34%, the group of pVAX1 was 20.92%, 41.8%, 43.78%, the group of pVAX1-ficolin-A was 13.05%, 28.78%, 36.15%, the group of pVAX1- MSP1_19_ was 3.72%, 21.05%, 33.36%, while the group of pVAX1-ficolin-A with pVAX1-MSP1_19_ was 3.16%, 15.85%, 22.23%. The results indicated ficolin-A can decrease Plasmodium invasion erythrocyte, but the effect of ficolin–A is less than MSP1-_19_. The percent of invasion sharply decrease when use ficolin-A and MSP1-_19_ stand together (Fig. 2).

### Analysis of survival rate

**Fig. 3:**
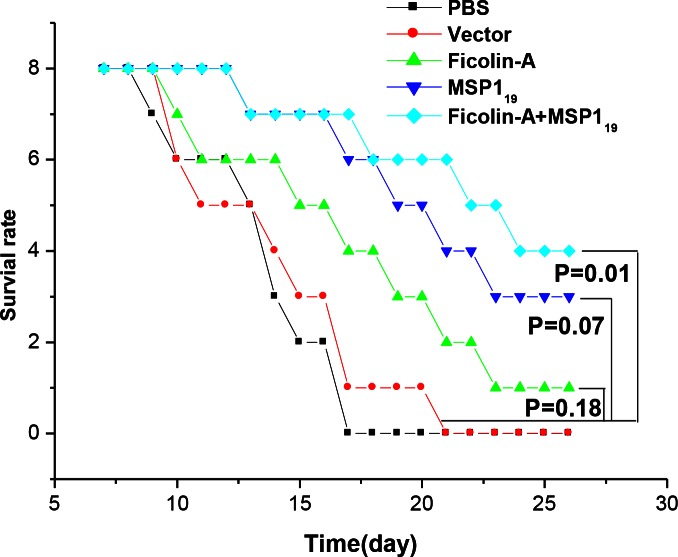
The survival rate was compared with the method of Kaplan-Meier (SPSS13.0), five groups were compared, PBS, pVAX1, pVAX1-Ficolin-A, pVAX1-MSP1_19_, pVAX1-ficolin-A and pVAX1- MSP1_19._

To observe the protection effect of ficolin-A, eight BALB/c mouse were sacrificed till 30 days after infection. The status was analyzed by Kaplan-Meier (SPSS 13.0). The results suggest both ficolin-A and MSP1_19_ haven’ significant effect of prolong survival time, compared with the group of pVAX1 (*P*=0.18, *P*=0.07). But the group of pVAX1-ficolin-A with pVAX1-MSP1_19_ has significantly prolong mice life compared with the group of pVAX1 (*P*=0.01). Although ficolin-A did not exhibit more protection than MSP1_19_, the data showed ficolin-A could enhance the protection of MSP1_19_ (Fig. 3).

## Discussion

Many factors are involved in this burden of malaria, and lack of an effective malaria vaccine (20). Highly purified protein antigens are usually poor immunogens, adjuvants are needed to obtain satisfactory immune responses. But thus induce draconic security issue for human being. Molecular from organism itself which could enhance candidate antigen immuprotection may overcome the problem. Some component of classics complement pathway such as C5a, were verified contribute to the pathogenesis of placental malaria (PM) by inducing dysregulated inflammatory and angiogenic response that impair placental function (21). And the oligomerization domain of C4-binding protein (C4bp) was reported that can play a new adjuvant-like effect, when it was fused to *P. yoelii* MSP_19_ and substantially increases the antigen's immunogenicity (22).

However, less is known about ficolin-A, whether play an active role in anti-malaria. Although there is no evidence show ficolin-A stimulate the innate immune system against malaria, our results indicated ficolin-A can enhance the immuoprotection of MSP1_19_ (Fig. 2; Fig. 3).

Since ficolins are normal component in organisms, they could be used as formulation in vaccine to avoid unsafe factor caused by adjuvant. It provides some evidence that human ficolins, such as L-ficolin and M-ficolin, can be used as available formulation in vaccine to defend the human infected *Plasmodium*. However, we need do more work to understand the anti-malaria mechanism of ficolin-A in mice, and whether excessive expression of ficolins in vivo might elicit other autoimmunity damages need further investigation.

## Conclusion

Our research suggest that ficolin-A can enhance the immunoprotection of MSP1_19_ in vivo, and ficolin-A may be used as immunoen-hancer in the study of vaccine defending malaria.
